# CYP2C19*2 and CYP2C19*17 variants and effect of tamoxifen on breast cancer recurrence: Analysis of the International Tamoxifen Pharmacogenomics Consortium dataset

**DOI:** 10.1038/s41598-017-08091-x

**Published:** 2017-08-10

**Authors:** Per Damkier, Anders Kjærsgaard, Kimberly A. Barker, Deidre Cronin-Fenton, Anatasha Crawford, Ylva Hellberg, Emilius A. M. Janssen, Carl Langefeld, Thomas P. Ahern, Timothy L. Lash

**Affiliations:** 1 Department of Clinical Biochemistry and Pharmacology, Odense University Hospital, Odense, Denmark; 2 Department of Clinical Research, University of Southern Denmark, Odense, Denmark; 3 Department of Clinical Epidemiology, Aarhus University, Aarhus, Denmark; 4 Department of Microbiology, Boston University School of Medicine, Boston, Massachusetts USA; 5 Department of Epidemiology, Rollins School of Public Health and Winship Cancer Institute, Emory University, Atlanta, GA USA; 6 Department of Pathology, Aarhus University Hospital, Aarhus, Denmark; 7 Department of Pathology, Stavanger University Hospital, Stavanger, Norway; 8 Center for Public Health Genomics and Department of Biostatistical Sciences, Wake Forest School of Medicine, Winston-Salem, North Carolina USA; 9 Departments of Surgery and Biochemistry, The Robert Larner, M.D. College of Medicine at The University of Vermont, Burlington, Vermont USA

## Abstract

The role of cytochrome P450 drug metabolizing enzymes in the efficacy of tamoxifen treatment of breast cancer is subject to substantial interest and controversy. CYP2D6 have been intensively studied, but the role of CYP2C19 is less elucidated, and we studied the association of CYPC19 genotype and recurrence of breast cancer. We used outcome and genotyping data from the large publicly available International Tamoxifen Pharmacogenomics Consortium (ITPC) dataset. Cox regression was used to compute the hazard ratios (HRs) for recurrence. CYP2C19 genotype data was available for 2 423 patients and the final sample cohort comprised 2 102 patients. CYP2C19*2 or *19 alleles did not influence DFS. For the CYP2C19*2 allele, the HR was 1.05 (CI 0.78–1.42) and 0.79 (CI 0.32–1.94) for hetero- and homozygote carriers, respectively. The corresponding HR for hetero- and homozygote carriers of the CYP2C19*17 allele were 1.02 (CI 0.71–1.46) and 0.57 (CI 0.26–1.24), respectively. Accounting for CYP2D6 genotype status did not change these estimates. We found no evidence to support a clinically meaningful role of CYP2C19 polymorphisms and response to tamoxifen in breast cancer patients and, consequently, CYP2C19 genotype status should not be included in clinical decisions on tamoxifen treatment.

## Introduction

Breast cancer is, excluding skin cancers, the most common malignancy among women in the United States and caused about 571.000 deaths world-wide in 2015^1, 2^. Tamoxifen is the standard treatment for premenopausal women with estrogen receptor (ER) positive breast cancer, and five years of adjuvant tamoxifen therapy reduces recurrences by nearly 50%^3–5^. In tumor cells, tamoxifen and its metabolites impede the binding of estrogen to the ER to inhibit expression of estrogen-responsive genes, thereby preventing tumor cell growth and angiogenesis^6, 7^. Patient responses to tamoxifen vary, and around 20–30% of patients receiving tamoxifen therapy in accordance with guidelines still suffer a breast cancer recurrence^8^.

**Figure 1:**
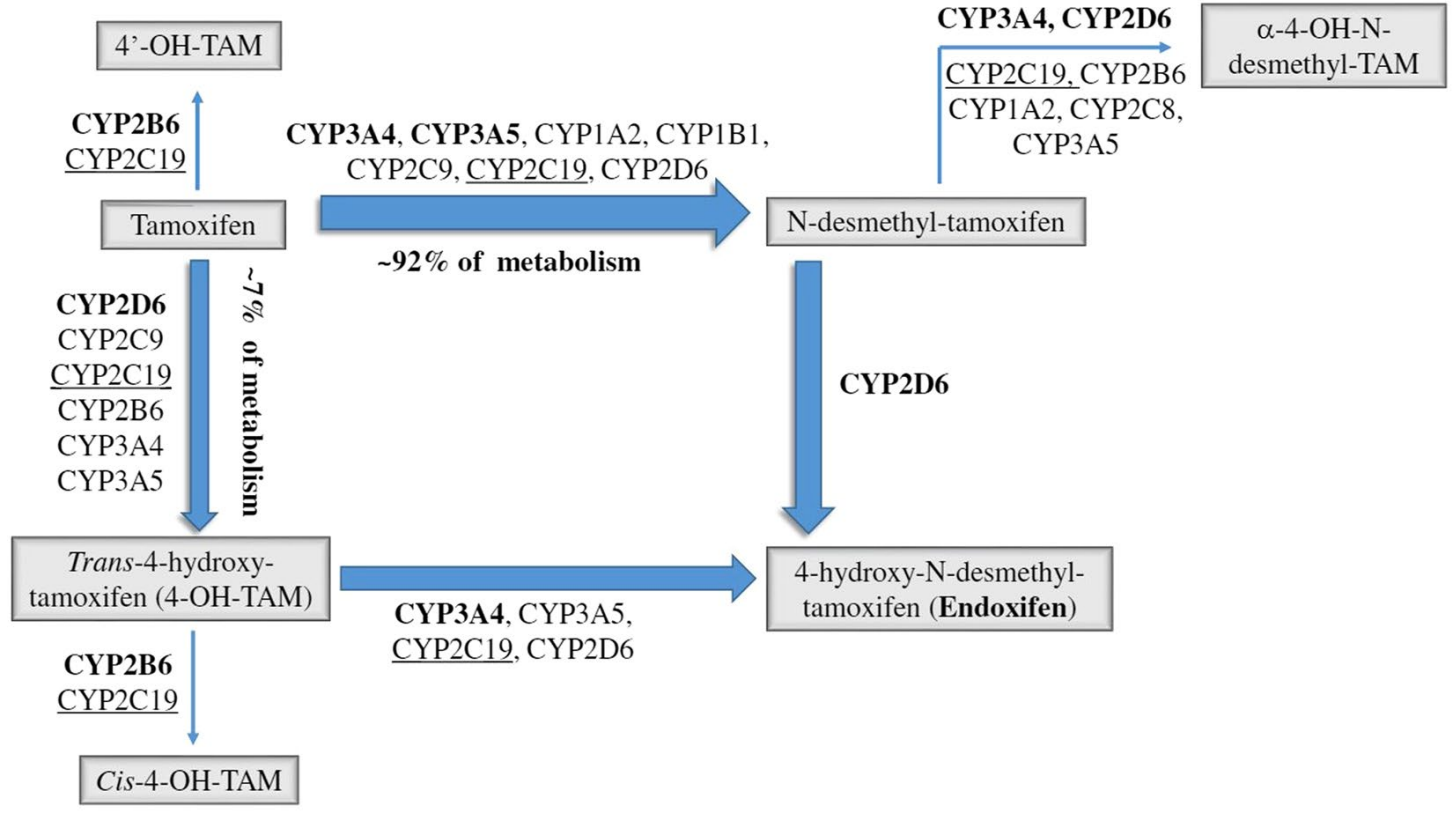
The metabolism of tamoxifen.

The complex tamoxifen metabolism (Fig. 1) is primarily catalyzed by cytochrome P450 (CYP) enzymes, which are subject to substantial differences in inter-individual expression and activity^9–12^. Endoxifen, the 4-hydroxy-N-desmethyl metabolite of tamoxifen, is central to mechanism of action and efficacy of tamoxifen, and concentrations thereof varies substantially between patients^6, 13, 14^. CYP2D6 is the principal enzyme catalyzing the conversion of tamoxifen to endoxifen, and the association of genomic variants in the *CYP2D6* gene to outcome of tamoxifen treatment has been extensively studied^15, 16^. Two of the largest datasets reported a null-association, but contradictory findings have led to ongoing controversy over the value of using *CYP2D6* genotyping to guide the prescription of tamoxifen^17–22^.

CYP2C19 catalyzes the formation of a proportion of tamoxifen metabolites, including the conversion of 4-OH-TAM to endoxifen (Fig. 1)^23^. The *CYP2C19* gene is highly polymorphic. Loss of enzyme activity results from the CYP2C19*2 (681 G > A, rs4244285)^24, 25^ and CYP2C19*3 (636 G > A, rs4986893) alleles^26, 27^. The *2 allele is found in approximately 23–39% of Asians, 10–20% of Caucasians, and 15% of Africans^23, 28^. The *3 allele occurs in 5–10% of Asians (17). CYP2C19*17 (−806C > T, rs12248560 or −3402C > T, rs11188072) has been implicated in enhanced gene transcription^26, 29, 30^. The *17 allele is found in about 4% of Asians and 18–24% of Caucasians and Africans^23, 28, 31, 32^.

Studies differ with respect to the associations observed between *CYP2C19* genotypes and clinical outcomes as well as to corresponding levels of tamoxifen and its metabolites^33–38^. Counterintuitively, the presence of a *2 allele has been associated with longer relapse-free time or better survival in tamoxifen-treated women in some^39–41^, but not all studies^32, 42–46^. In some studies the CYP2C19*17 allele is associated with more favorable outcomes in breast cancer patients treated with tamoxifen^32^, though null results have also been found^40, 42, 43, 47^. Contradictory results were obtained in the context of tamoxifen monotherapy in advanced breast cancer, where an association between the *17 allele and shorter time to treatment failure has been reported^39, 41^.

Given these contradictory findings, we used a large, publicly available dataset to investigate the association of CYP2C19*2 and CYP2C19*17 variants with breast cancer recurrence in both pre- and postmenopausal women treated with adjuvant tamoxifen therapy for ER-positive breast cancer.

## Subjects and Methods

### Data source and study population

**Figure 2:**
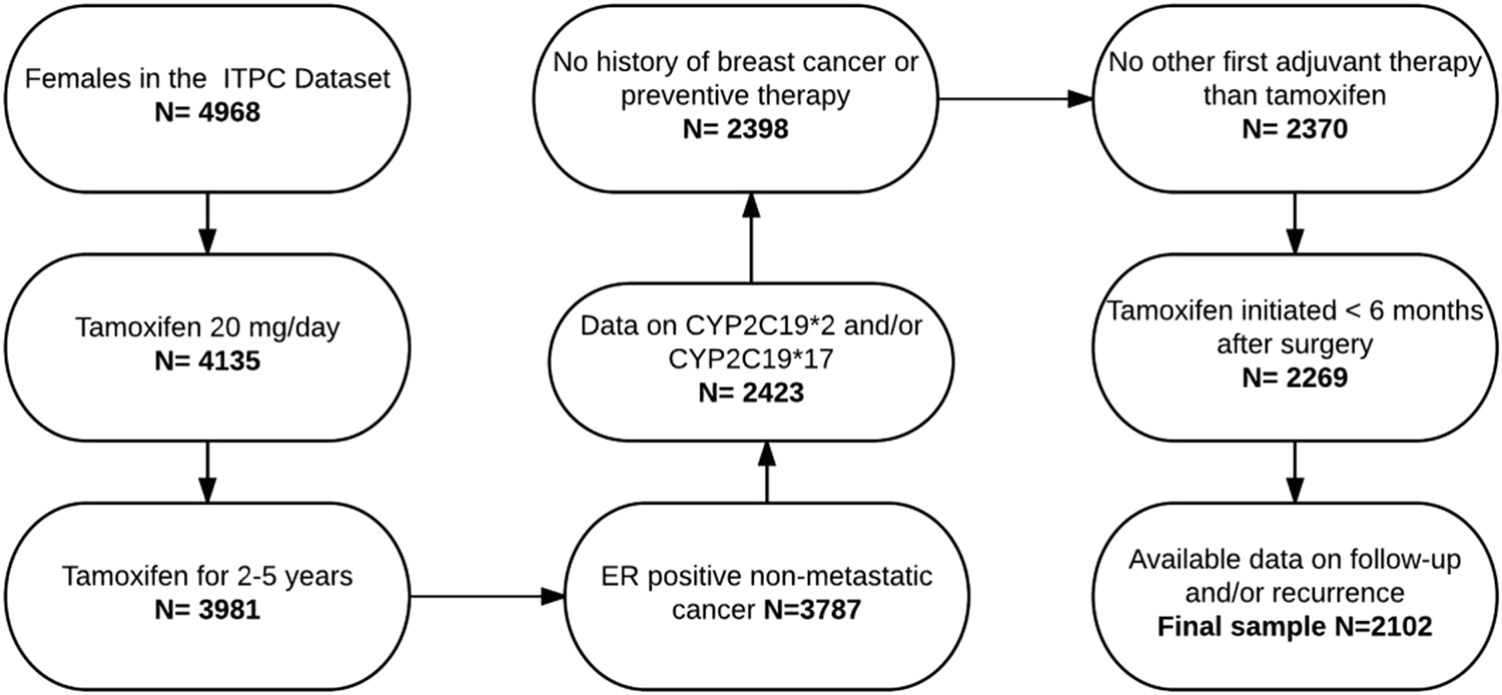
Study population flowchart.

The ITPC comprises research from 12 sites representing 9 countries, all designed to prospectively assess the contribution of genetic variation in tamoxifen metabolism and transport pathways to breast cancer recurrence risk. We required that patients had been prescribed 20 mg/day tamoxifen for an intended duration of 2 or 5 years, had not previously received systemic therapy for breast cancer prevention, had no known history of invasive or *in situ* breast cancer, had not used any other adjuvant therapy before tamoxifen, and initiated tamoxifen therapy within 182 days of breast cancer surgery. We included patients with non-metastatic, ER-positive tumors who had data on at least one *CYP2C19* variant, whether a recurrence occurred, and follow-up time (Fig. 2).

### Analytic variables

Disease-free survival time (DFS) was the number of months from diagnosis until breast cancer recurrence, defined as an ipsilateral local or regional recurrence (invasive or non-invasive), a distant recurrence, or a contralateral breast cancer (invasive or non-invasive). Patients who did not experience a recurrence were censored on the date of death from another cause or on the day of last disease-free evaluation.

Genotype exposures were CYP2C19*2 and CYP2C19*17, with reference to the wild-type CYP2C19*1. Various methods of genotyping were used in the seven studies comprising the data, with the majority of genotypes (60.5%) ascertained by the AmpliChip test platform (Roche Molecular Diagnostics, California, USA). In three instances where multiple methods were used for a single individual and the AmpliChip blood genotype did not match the CYP2C19*2 genotype obtained with another method, preference was given to the AmpliChip data due to the high sensitivity and specificity of this test^49^. The CYP2C19*3 allele was not assessed in this study because no variants were detected in the included data.

### Covariables

Potential covariates of interest were: age, ethnicity, menopausal status, tumor grade and stage, progesterone receptor (PR) status, use of other adjuvant therapies (radiation and chemotherapy), and CYP2D6 metabolizer phenotype. Age (as a continuous variable), menopausal status (pre-, post-, or peri-), PR status, use of other adjuvant therapies, and Nottingham tumor grades were recorded directly in the ITPC dataset. Perimenopausal women (n = 57) were combined with post-menopausal women for all analyses. Categories as defined by the Office of Management and Budget^50^ were used to divide patients into three ethnic groups: Caucasian, Asian or Pacific Islander, and any other ethnicity (which included African-Americans, mixed ethnicity individuals, and individuals of any other ethnicity).

Tumor stage was derived from information on both tumor diameter and the number of positive lymph nodes. Missing information on *in situ* tumors and distant metastases prohibited use of the TNM staging system; however, the primary tumor and pathologic guidelines of the TNM system were used to classify tumors into five stages^51^.

A variable encoding individuals’ CYP2D6 metabolizer phenotype (ultra- UM, extensive- EM, intermediate- IM, or poor- PM) was available in the ITPC data, and accounted for both genetic factors and the use of CYP2D6-inhibiting drugs. We generated a variable designating overall tamoxifen metabolic activity (high, intermediate or low) by combining CYP2D6 phenotypes and *CYP2C19* genotypes according to Schroth (Table 1)^32^.

**Table 1:** Phenotype assignment according to CYP2D6 and CYP2C19 genotype.

Inferred phenotype levels of combined CYP2D6 phenotypes^1^ and CYP2C19*17 genotypes		
Level	CYP2D6 phenotype	CYP2C19*17 allele presence
**1**	EM/EM	Yes
**2**	EM/EM	No
	EM/IM	Yes
	EM/PM	Yes
**3**	EM/IM	No
	EM/PM	No
	IM/PM	Yes or No
	IM/IM	Yes or No
	PM/PM	Yes or No
Inferred phenotype levels of combined CYP2D6 phenotypes^1^ and CYP2C19*2 genotypes		
Level	CYP2D6 phenotype	CYP2C19*2 allele presence
**1**	EM/EM	No
**2**	EM/EM	Yes
	EM/IM	No
	EM/PM	No
**3**	EM/IM	Yes
	EM/PM	Yes
	IM/PM	Yes or No
	IM/IM	Yes or No
	PM/PM	Yes or No

^1^CYP2D6 UM considered as EM for creation of these levels.

### Statistical analyses

Descriptive analyses including all covariates of interest were computed for all women analyzed. Cox regression was used to compute the hazard ratios (HRs) for recurrence and associated 95% confidence intervals (CIs). The tumor grade variable violated the proportional hazards assumption when assessed using log-log survival curves and were therefore excluded from all models. Models containing all possible variable subsets were analyzed using the change-in-estimate approach, with confounding indicated in models where the variable subset removed led to a hazard ratio changed by greater than 10% compared with the hazard ratio for the full model^52^. Final Cox proportional hazards models included age at diagnosis of primary breast cancer (as a continuous variable), tumor stage, and ethnicity (Caucasian or Asian, for CYP2C19*2 only) as covariates. Supplemental analyses stratified by CYP2D6 phenotype and menopausal status were also performed.

For multivariable analyses, individuals with missing values for any modeled variable were excluded. To assess the potential for bias due to the use of complete case analyses, imputation of missing values for CYP2C19*2 genotype, CYP2C19*17 genotype, ethnicity, age at breast cancer diagnosis, and tumor stage was done in a supplemental analysis.

All analyses were carried out in SAS version 9.4 (Cary, NC).

### Data availability statement

Data were obtained from the International Tamoxifen Pharmacogenomics Consortium (ITPC) which are publicly available^48^.

## Results

### Study population

The seven sites containing eligible patients provided 2 102 women for analysis (Fig. 2). Of these, 296 women experienced a breast cancer recurrence. One woman who did not have a recurrence and was missing data on the last disease-free evaluation was censored on the date she was last known to be alive. Patient characteristics for the sample and source population by study site are presented in Table 2 and Supplementary Table S1, respectively. Characteristics of the sample and source data stratified by recurrence are presented in Supplementary Table S2. The median DFS was 61 months for all women, 45 months for women experiencing a recurrence, and 63 months for women with no recurrence.

**Table 2:** Sample population characteristics.

Total	N	Project site							Total
		2	4	6	8	9	11	12	
		186	217	191	875	73	305	255	2102
**Median DFS in months (range)**	Median	45	52	124	64	28	65	68	61
	Min	7.2	1.2	3.1	2.1	7.1	0.33	4.2	0.33
	Max	173	81	207	244	132	140	121	244
**Age at diagnosis**	Mean	54	51	61	65	48	62	45	59
	SD	11	10	10	9.8	10	14	8	13
**Recurrence**									
No	N	141	214	123	756	71	262	239	1806
	%	75.8	98.6	64.4	86.4	97.3	85.9	93.7	85.9
Yes	N	45	3	68	119	2	43	16	296
	%	24.2	1.4	35.6	13.6	2.7	14.1	6.3	14.1
**Menopausal status at diagnosis**									
Missing	N	4	4	40	0	0	20	208	276
	%	2.2	1.8	20.9	0	0	6.6	81.6	13.1
Premenopausal	N	78	64	10	43	0	66	0	261
	%	41.9	29.5	5.2	4.9	0	21.6	0	12.4
Postmenopausal	N	104	149	141	832	73	219	47	1565
	%	55.9	68.7	73.8	95.1	100	71.8	18.4	74.5
CYP2C19*2 genotype									
Missing or unknown	N	3	1	0	43	0	0	0	47
	%	1.6	0.5	0	4.9	0	0	0	2.2
Wild type	N	124	151	140	586	51	226	139	1417
	%	66.7	69.6	73.3	67	69.9	74.1	54.5	67.4
One null function allele	N	57	61	48	223	17	75	86	567
	%	30.6	28.1	25.1	25.5	23.3	24.6	33.7	27
Two null function alleles	N	2	4	3	23	5	4	30	71
	%	1.1	1.8	1.6	2.6	6.8	1.3	11.8	3.4
CYP2C19*17 genotype									
Missing or unknown	N	0	0	191	25	73	305	255	849
	%	0	0	100	2.9	100	100	100	40.4
Wild type	N	107	133	0	489	0	0	0	729
	%	57.5	61.3	0	55.9	0	0	0	34.7
One gain of function allele	N	73	78	0	294	0	0	0	445
	%	39.2	35.9	0	33.6	0	0	0	21.2
Two gain of function alleles	N	6	6	0	67	0	0	0	79
	%	3.2	2.8	0	7.7	0	0	0	3.8

### Genotypes

Data on CYP2C19*2 were available from all seven study sites for 2 055 women, and data on CYP2C19*17 were reported from three sites for 1 253 women. Distributions and Hardy-Weinberg chi-squared statistics within each study site for the CYP2C19*2 and CYP2C19*17 genotypes and DNA sources are provided for sample and source populations in Supplementary Tables S3 and S4. Both variants were in Hardy-Weinberg equilibrium for each study site, except for CYP2C19*17 at site 8 (p = 0.02) and CYP2D6*2 at site 12 (p = 0.005)

### CYP2C19 genotypes and DFS

For the CYP2C19*2 allele, adjusted hazard ratios for associations between variant heterozygotes and homozygotes with DFS were 1.05 (95% CI: 0.78, 1.42) and 0.79 (95% CI: 0.32, 1.94), respectively (Table 3). For the CYP2C19*17 allele, adjusted hazard ratios for associations between variant heterozygotes and homozygotes with DFS were 1.02 (95% CI: 0.71, 1.46) and 0.57 (0.26, 1.24), respectively (Table 3). Stratification by menopausal status and CYP2D6 phenotype did not yield any notable associations between *CYP2C19* genotype and DFS (Supplementary Table S5). Results based on imputed data sets were not substantially different from the complete case analysis, but in general tended to be closer to the null (Supplementary Table S6).

**Table 3:** Cox proportional hazard ratios for CYP2C19*2 and CYP2C19*17 genotypes and Disease Free Survival.

Comparison	Hazard Ratio (95% CI); (N)
CYP2C19*2	
*Genotypes*	
No *2 allele	1.0 (Reference); (971)
*2/*1	1.05 (0.78–1.42); (420)
*2/*2	0.79 (0.32–1.94); (54)
CYP2C19*17	
*Genotypes*	
No *17 allele	1.0 (Reference); (609)
*17/*1	1.02 (0.71–1.46); (361)
*17/*17	0.57 (0.26–1.24); (71)

### CYP2D6 phenotype/CYP2C19 genotype combinations

Results are provided in Table 4. For the CYP2D6 phenotype/CYP2C19*2 genotype combinations, multivariate DFS hazard ratios for the phenotypically designated “high” and “intermediate” tamoxifen metabolic activity groups were 0.86 (95% CI: 0.45–1.66) and 0.89 (95% CI: 0.46–1.74), respectively, compared with the “low” metabolic activity group. The corresponding adjusted DFS hazard ratios for the CYP2D6 phenotype/CYP2C19*17 genotype combinations were 1.19 (95% CI: 0.73–1.94) and 1.21 (95% CI: 0.80–1.85).

**Table 4:** Cox proportional hazard ratios for combinations of CYP2D6 metabolizer phenotypes with CYP2C19*2 or CYP2C19*17 genotypes and Disease Free Survival.

Comparison	Hazard Ratio (95% CI); (N)
CYP2C19*2	
Level^1^ 3	1.0 (Reference); (85)
Level 2	0.89 (0.46–1.74); (539)
Level 1	0.86 (0.45–1.66); (836)
CYP2C19*17	
Level 3	1.0 (Reference); (230)
Level 2	1.21 (0.80–1.85); (1158)
Level 1	1.19 (0.73–1.94); (376)

^1^Levels are defined in Table 1.

## Discussion

We found no evidence of a clinically meaningful association between CYP2C19*2 or CYP2C19*17 genotypes and DFS in tamoxifen-treated breast cancer patients in a large dataset. A secondary analysis of *CYP2C19* genotype accounting for CYP2D6 phenotypes resulted in little change to the observation.

This study has the largest overall sample size of work on this topic to date and includes a larger number of *CYP2C19* variants compared with prior studies. Even so, our estimates come with confidence intervals that suggest some limitation with respect to sample-size This study also benefits from the inclusion of a substantial number of premenopausal patients, permitting stratification of the association by menopausal status. Only two other studies have examined the association between *CYP2C19* genotype and breast cancer recurrence within strata of menopausal status^42, 44^, and those studies included a combined total of only 85 premenopausal patients. The inclusion of a large premenopausal cohort is especially relevant as tamoxifen is the guideline endocrine therapy for these women^5^.

Combining CYP2D6 and CYP2C19*2 or CYP2C19*17 into singular phenotypes (as suggested by Schroth *et al*.^32^) did not suggest that this is of clinical relevance (Table 4). While this finding should be interpreted with caution, as confidence intervals are somewhat wide, this analysis indirectly lends further weight against the heavily discussed clinically meaningful role of CYP2D6 itself. Our results differ somewhat from those reported by Schroth, who reported a statistically significant inference of the CYP2C19*17 allele on event-free survival^32^. Our sample size is several orders of magnitude larger though, which we believe explains this apparent discrepancy.

At ITPC sites not testing for the CYP2C19*17 allele, misclassification of tamoxifen metabolic phenotype could have occurred, but a stratified sensitivity analysis restricted to sites testing for CYP2C19*17 did not provide substantially different results. CYP2C19*3 allele misclassification is unlikely to influence the overall result as this allele would only be expected to be common at site 12^23^. Allele distribution was reasonably consistent across study sites and compared well to reported literature frequencies. This suggest that errors from genotyping are less likely to present a main issue within our dataset. The CYP2C19*2 allele was assessed across 10 study sites. The allele frequencies were reasonably comparable, 23–31% for heterozygosity, bar two sites (project site #6 and #12) which yielded frequencies of 18 and 35% for *2 heterozygosity, respectively. These frequencies are within reason of the expected given the variability related to sample size and ethnicity composition of the respective populations. Site 12 had a relative low sample size and site 6 a high degree of missing values (32%). The latter diluted the frequency as, among those tested, 26% were heterozygous for the *2 allele. The CYP2C19*17 was only assessed at three sites that yielded homogenous and comparable allele frequencies with one gain of function allele frequencies between 32–37%. Allele distributions per ethnicity (Supplementary Table S5) compared well to reported literature frequencies, though we could not meaningfully compare the *17 allele frequency in Asian subjects to literature data as very few of Asian origin were tested in our sample. The lack of data on CYP2C19 inhibitor use could have biased our estimates towards the null. *CYP2C19* genotyping using tumor-derived DNA (at three sites) may introduce misclassification due to potential loss-of-heterozygosity in tumor cells^53, 54^.Results of chi-squared tests for Hardy-Weinberg equilibrium indicate that loss-of-heterozygosity had a minor impact on observed *CYP2C19* genotypes in this study. A minor violation of HWE was observed at study site 8, which accounted for the majority of samples assessed for CYP2C19*17. This minor violation represents a weakness even if misclassification due to loss-of-heterozygosity appears less likely to result in significant bias of overall study estimates^20^. A violation of HWE for CYP2C19*2 was observed at study site 12, but the sample of 240 subjects contributed little to the overall analysis.

While previous reports have found the presence of CYP2C19*2 to be associated with superior efficacy of tamoxifen treatment^39–41^, our results support other studies reporting no such association^32, 42–46^. The hazard ratio for the association of CYP2C19*17 homozygotes with a favorable DFS (HR = 0.57, 95% CI: 0.26, 1.24) is similar to the ratio found previously for the association of carrying CYP2C19*17 with relapse-free time (HR = 0.45, 95% CI: 0.21, 0.92) (31). About 40% of the patient population and the majority of *17 allele data in the ITPC dataset were from the latter study, so our study should not be viewed as independent evidence. Our findings for the *17 allele are consistent with results from a smaller, similar study, which reported a hazard ratio of 0.93 (95% CI = 0.64, 1.37) and found a near-null association among those with impaired CYP2D6^47^. Despite the biologic plausibility of *CYP2C19* playing an important role in patients with reduced CYP2D6 function, our stratified analyses do not support this hypothesis. The complex metabolism of tamoxifen, which include catalytic activity of CYP2C19, CYP1A2, CYP3A4/5, CYP2D6, CYP2B6 and CYP2C9, may explain the null-association found in this study. The formation of active tamoxifen metabolites in patients carrying reduced or increased CYP2C19 function alleles may be sufficiently compensated through parallel and serial metabolic pathways catalyzed by other P450 enzymes. This would mitigate the net overall clinical consequence of genomic CYP2C19 variants and result in a statistical inference toward the null.

A key limitation is that the ITPC dataset does not allow for differentiation between predictive and prognostic markers, since studies did not include women diagnosed with ER-negative tumors who were not treated with tamoxifen. Several studies indicate that *CYP2C19* variants are associated with differences in baseline breast cancer risk, likely due to the inherent role of *CYP2C19* in the metabolism of estrogen. However, this association has not been consistently observed, and the fact that the minor allele frequencies observed here match population-wide benchmarks argues against *CYP2C19* genotype as a selection force. On the other hand, breast cancer etiology or survival is usually only relevant after childbirth in most women, which would render selection pressure less relevant.

Province *et al*. analyzed the ITPC dataset and reported poorer disease-free survival among CYP2D6 poor metabolizers and a weak association between poor metabolizer status and a shorter breast cancer-free interval^15^. These associations were not robust to variations in inclusion criteria, and this study has been heavily criticized for its reliance on statistical interpretations of *ad hoc* subset analyses and this issue remains highly controversial^55–58^. In light of these criticisms, the criteria for inclusion in our study were defined *a priori*. Province *et al*. also described the heterogeneity of results between the study sites, which is an additional challenge in interpreting the results of our study^15^.

In conclusion, we found no evidence to support a clinically meaningful role of CYP2C19 polymorphisms and response to tamoxifen in breast cancer patients. Given the complexity of tamoxifen pharmacodynamics and metabolism and the divergent results on the importance of genomic variants, it appears unlikely that a clinically useful simple predictive set of genomic variables will be identified.

## Electronic supplementary material

Supplementary information
