# Supplementary material for: CYP2C19*2 and CYP2C19*17 variants and effect of tamoxifen on breast cancer recurrence: Analysis of the International Tamoxifen Pharmacogenomics Consortium dataset
Source: Sci Rep. 2017 Aug 10;7:7727. doi: 10.1038/s41598-017-08091-x (PMC5552748; doi:10.1038/s41598-017-08091-x)
Supplement: Supplementary file 1 — Supplementary information [file 41598_2017_8091_MOESM1_ESM.pdf]

Title

CYP2C19*2 and CYP2C19*17 variants and effect of tamoxifen on breast cancer recurrence: Analysis of the International Tamoxifen Pharmacogenomics Consortium dataset.

Authors and affiliations

Per Damkier, MD[1, 2]

Anders Kjærsgaard, PhD[3]

Kimberly A. Barker, PhD[4]

Deidre Cronin-Fenton, PhD [3]

Anatasha Crawford, PhD[5]

Ylva Hellberg, MD[6]

Emilius A.M. Janssen, PhD[7]

Carl Langefeld, PhD[8]

Thomas P. Ahern, PhD[9]

Timothy L. Lash, PhD[3, 5]

1. Department of Clinical Biochemistry and Pharmacology, Odense University Hospital, Odense, Denmark

2. Department of Clinical Research, University of Southern Denmark, Odense, Denmark

3. Department of Clinical Epidemiology, Aarhus University, Aarhus, Denmark

4. Department of Microbiology, Boston University School of Medicine, Boston, Massachusetts, USA

5. Department of Epidemiology, Rollins School of Public Health and Winship Cancer Institute, Emory University, Atlanta, GA

6. Department of Pathology, Aarhus University Hospital, Aarhus, Denmark

7. Department of Pathology, Stavanger University Hospital, Stavanger, Norway

8. Center for Public Health Genomics and Department of Biostatistical Sciences, Wake Forest School of Medicine, Winston-Salem, North Carolina, USA

9. Departments of Surgery and Biochemistry, The Robert Larner, M.D. College of Medicine at The University of Vermont, Burlington, Vermont, USA

Author for correspondence

Per Damkier

Department of Clinical Biochemistry and Pharmacology

Odense University Hospital

DK5000 Odense

Denmark

Mail: pdamkier@health.sdu.dk

Supplementary Table S1

Source population characteristics

		Project site												
		1	2	3	4	5	6	7	8	9	10	11	12	**Total**
**Total**	N	174	320	282	267	214	423	801	1140	165	519	398	270	4973
**Median DFS in months (range)**	Median	52	39	78	52	133	114	95	66	26	79	63	68	68
	Min	3.2	7.2	7.1	1.2	1.4	3.1	0.49	1.3	6.1	0.03	0.10	4.2	0.03
	Max	166	173	118	81	202	406	224	244	132	124	143	121	406
**Age at diagnosis**	Mean	58	55	54	51	68	58	67	66	46	59	60	45	60
	SD	12	12	11	10	8.3	14	10	9.9	9.3	12	14	7.9	13
**Recurrence**														
Unknown	N	0	0	0	0	0	5	244	0	23	0	0	0	272
	%	0	0	0	0	0	1.2	30.5	0	13.9	0	0	0	5.5
No	N	143	243	240	262	139	239	344	948	139	405	339	249	3690
	%	82.2	75.9	85.1	98.1	65.0	56.5	42.9	83.2	84.2	78.0	85.2	92.2	74.2
Yes	N	31	77	42	5	75	179	213	192	3	114	59	21	1011
	%	17.8	24.1	14.9	1.9	35.0	42.3	26.6	16.8	1.8	22.0	14.8	7.8	20.3
**Menopausal status at diagnosis**														
Missing	N	39	9	10	4	0	167	0	0	0	519	28	220	996
	%	22.4	2.8	3.5	1.5	0	39.5	0	0	0	100	7.0	81.5	20.0
Premenopausal	N	37	130	123	83	0	18	0	53	55	0	109	0	608
	%	21.3	40.6	43.6	31.1	0	4.3	0	4.6	33.3	0	27.4	0	12.2
Postmenopausal	N	98	181	149	180	214	238	801	1087	110	0	261	50	3369
	%	56.3	56.6	52.8	67.4	100.0	56.3	100.0	95.4	66.7	0	65.6	18.5	67.7
**CYP2C19*2 genotype**														
Missing or unknown	N	0	5	282	7	0	135	801	150	0	0	0	0	1380
	%	0	1.6	100	2.6	0	31.9	100	13.2	0	0	0	0	27.7
Wild type	N	174	210	0	188	214	209	0	702	105	519	291	145	2757
	%	100	65.6	0	70.4	100	49.4	0	61.6	63.6	100	73.1	53.7	55.4
One null function allele	N	0	98	0	68	0	74	0	261	50	0	100	94	745
	%	0	30.6	0	25.5	0	17.5	0	22.9	30.3	0	25.1	34.8	15.0
Two null function alleles	N	0	7	0	4	0	5	0	27	10	0	7	31	91
	%	0	2.2	0	1.5	0	1.2	0	2.4	6.1	0	1.8	11.5	1.8
**CYP2C19*17 genotype**														
Missing or unknown	N	174	0	282	6	214	423	801	58	165	519	398	270	3310
	%	100	0	100	2.2	100	100	100	5.1	100	100	100	100	66.6
Wild type	N	0	194	0	163	0	0	0	630	0	0	0	0	987
	%	0	60.6	0	61.0	0	0	0	55.3	0	0	0	0	19.8
One gain of function allele	N	0	117	0	92	0	0	0	364	0	0	0	0	573
	%	0	36.6	0	34.5	0	0	0	31.9	0	0	0	0	11.5
Two gain of function alleles	N	0	9	0	6	0	0	0	88	0	0	0	0	103
	%	0	2.8	0	2.2	0	0	0	7.7	0	0	0	0	2.1

Supplementary Table S2

Source and sample population characteristics stratified by recurrence

		Sample population characteristics	Recurrence		Source population characteristics	Recurrence		
			No	Yes		Unknown	No	Yes
**Total**	N	2 102	1 806	296	4 973	272	3 690	1 011
**Age at diagnosis**	Mean	59	59	61	60	71	59	61
	SD	13	13	12	13	13	13	13
**Ethnicity**								
Unknown	N	308	265	43	1 218	244	695	279
	%	14.7	14.7	14.5	24.5	89.7	18.8	27.6
White	N	1 506	1 271	235	3 103	23	2 425	655
	%	71.6	70.4	79.4	62.4	8.5	65.7	64.8
Black	N	11	11	0	52	0	41	11
	%	0.5	0.6	0	1.0	0	1.1	1.1
Asian or Pacific islander	N	276	258	18	598	5	527	66
	%	13.1	14.3	6.1	12.0	1.8	14.3	6.5
Mixed or other	N	1	1	0	2	0	2	0
	%	0	0.1	0	0	0	0.1	0
**Menopausal status at diagnosis**								
Missing	N	276	246	30	996	1	777	218
	%	13.1	13.6	10.1	20.0	0.4	21.1	21.6
Premenopausal	N	261	227	34	608	10	518	80
	%	12.4	12.6	11.5	12.2	3.7	14.0	7.9
Postmenopausal	N	1 565	1 333	232	3 369	261	2 395	713
	%	74.5	73.8	78.4	67.7	96.0	64.9	70.5
**Tumor Nottingham grade**								
Missing	N	407	366	41	1 685	268	1 081	336
	%	19.4	20.3	13.9	33.9	98.5	29.3	33.2
I	N	263	248	15	458	0	409	49
	%	12.5	13.7	5.1	9.2	0	11.1	4.8
II	N	1 078	936	142	1 982	4	1 601	377
	%	51.3	51.8	48.0	39.9	1.5	43.4	37.3
III	N	354	256	98	848	0	599	249
	%	16.8	14.2	33.1	17.1	0	16.2	24.6
**Tumor stage at diagnosis**								
Unknown	N	262	213	49	990	49	708	233
	%	12.5	11.8	16.6	19.9	18.0	19.2	23.0
I	N	810	753	57	1341	2	1197	142
	%	38.5	41.7	19.3	27.0	0.7	32.4	14.0
IIa	N	605	523	82	1 439	117	1 081	241
	%	28.8	29.0	27.7	28.9	43.0	29.3	23.8
IIb	N	224	177	47	578	53	384	141
	%	10.7	9.8	15.9	11.6	19.5	10.4	13.9
IIIa	N	146	106	40	436	34	248	154

		Sample population characteristics	Recurrence		Source population characteristics	Recurrence		
			No	Yes		Unknown	No	Yes
	%	6.9	5.9	13.5	8.8	12.5	6.7	15.2
IIIb	N	55	34	21	189	17	72	100
	%	2.6	1.9	7.1	3.8	6.3	2.0	9.9
**Progesterone receptor status**								
Unknown	N	61	59	2	636	23	420	193
	%	2.9	3.3	0.7	12.8	8.5	11.4	19.1
Negative	N	287	233	54	677	33	463	181
	%	13.7	12.9	18.2	13.6	12.1	12.5	17.9
Positive	N	1 754	1 514	240	3 660	216	2 807	637
	%	83.4	83.8	81.1	73.6	79.4	76.1	63.0
**Radiation treatment**								
Unknown	N	164	157	7	563	81	420	62
	%	7.8	8.7	2.4	11.3	29.8	11.4	6.1
No	N	664	564	100	1527	77	1 084	366
	%	31.6	31.2	33.8	30.7	28.3	29.4	36.2
Yes	N	1 274	1 085	189	2 883	114	2 186	583
	%	60.6	60.1	63.9	58.0	41.9	59.2	57.7
**Chemotherapy**								
Unknown	N	27	26	1	104	22	75	7
	%	1.3	1.4	0.3	2.1	8.1	2.0	0.7
No	N	1 646	1 411	235	3 885	241	2 858	786
	%	78.3	78.1	79.4	78.1	88.6	77.5	77.7
Yes	N	429	369	60	984	9	757	218
	%	20.4	20.4	20.3	19.8	3.3	20.5	21.6
**CYP2D6 phenotype**								
Unknown	N	33	30	3	96	5	74	17
	%	1.6	1.7	1.0	1.9	1.8	2.0	1.7
Extensive metabolizer	N	1 744	1 491	253	4 267	243	3 138	886
	%	83.0	82.6	85.5	85.8	89.3	85.0	87.6
Intermediate metabolizer	N	209	185	24	366	3	300	63
	%	9.9	10.2	8.1	7.4	1.1	8.1	6.2
Poor metabolizer	N	116	100	16	244	21	178	45
	%	5.5	5.5	5.4	4.9	7.7	4.8	4.5
**CYP2C19*2 genotype**								
Missing or unknown	N	47	43	4	1 380	246	801	333
	%	2.2	2.4	1.4	27.7	90.4	21.7	32.9
Wild type	N	1 417	1 215	202	2 757	12	2 205	540
	%	67.4	67.3	68.2	55.4	4.4	59.8	53.4
One null function allele	N	567	486	81	745	10	611	124
	%	27.0	26.9	27.4	15.0	3.7	16.6	12.3
Two null function alleles	N	71	62	9	91	4	73	14
	%	3.4	3.4	3.0	1.8	1.5	2.0	1.4
**CYP2C19*17 genotype**								
Missing or unknown	N	849	717	132	3310	272	2 294	744
	%	40.4	39.7	44.6	66.6	100.0	62.2	73.6
Wild type	N	729	632	97	987	0	821	166

		Sample population characteristics	Recurrence		Source population characteristics	Recurrence		
			No	Yes		Unknown	No	Yes
	%	34.7	35.0	32.8	19.8	0	22.2	16.4
One gain of function allele	N	445	387	58	573	0	490	83
	%	21.2	21.4	19.6	11.5	0	13.3	8.2
Two gain of function alleles	N	79	70	9	103	0	85	18
	%	3.8	3.9	3.0	2.1	0	2.3	1.8

Supplementary Table S3

Sample population DNA source

		Project site							
		2	4	6	8	9	11	12	**Total**
**Source of *2 tumor DNA**									
Missing or unknown	N	0	0	0	0	73	0	0	73
	%	0	0	0	0	100	0	0	3.5
Fresh frozen plasma	N	0	0	0	381	0	0	0	381
	%	0	0	0	43.5	0	0	0	18.1
Blood	N	186	217	0	470	0	0	255	1 128
	%	100	100	0	53.7	0	0	100	53.7
Fresh frozen tumor tissue	N	0	0	191	24	0	305	0	520
	%	0	0	100	2.7	0	100	0	24.7
**Source of *17 tumor DNA**									
Missing or unknown	N	0	9	191	0	73	305	0	578
	%	0	4.1	100	0	100	100	0	27.5
Fresh frozen plasma	N	0	0	0	381	0	0	0	381
	%	0	0	0	43.5	0	0	0	18.1
Blood	N	186	208	0	470	0	0	255	1 119
	%	100	95.9	0	53.7	0	0	100	53.2
Fresh frozen tumor tissue	N	0	0	0	24	0	0	0	24
	%	0	0	0	2.7	0	0	0	1.1

Supplementary Table S4

Source population DNA source and Hardy-Weinberg Equilibrium for CYP2C19 alleles

		Project site												
		1	2	3	4	5	6	7	8	9	10	11	12	**Total**
**Total**	N	174	320	282	267	214	423	801	1 140	165	519	398	270	4 973
**Source of *2 tumor DNA**														
Missing or unknown	N	174	0	282	0	214	115	801	0	165	519	0	0	2 270
	%	100	0	100	0	100	27.2	100	0	100	100	0	0	45.6
Fresh frozen plasma	N	0	0	0	0	0	0	0	445	0	0	0	0	445
	%	0	0	0	0	0	0	0	39.0	0	0	0	0	8.9
Blood	N	0	320	0	267	0	0	0	594	0	0	0	270	1 451
	%	0	100	0	100	0	0	0	52.1	0	0	0	100	29.2
Fresh frozen tumor tissue	N	0	0	0	0	0	308	0	101	0	0	398	0	807
	%	0	0	0	0	0	72.8	0	8.9	0	0	100	0	16.2
**Source of *17 tumor DNA**														
Missing or unknown	N	174	0	282	16	214	423	801	0	165	519	398	0	2 992
	%	100	0	100	6.0	100	100	100	0	100	100	100	0	60.2
Fresh frozen plasma	N	0	0	0	0	0	0	0	445	0	0	0	0	445
	%	0	0	0	0	0	0	0	39.0	0	0	0	0	8.9
Blood	N	0	320	0	251	0	0	0	594	0	0	0	270	1 435
	%	0	100	0	94.0	0	0	0	52.1	0	0	0	100	28.9
Fresh frozen tumor tissue	N	0	0	0	0	0	0	0	101	0	0	0	0	101
	%	0	0	0	0	0	0	0	8.9	0	0	0	0	2.0
**Hardy-Weinberg Equilibrium**														
*2 allele	$\chi2$	-	2.69	-	0.59	-	0.24	-	0.10	3.78	-	0.64	7.78	
	P		0.10		0.44		0.63		0.75	0.05		0.42	0.005	
*17 allele	$\chi2$	-	2.38	-	1.87	-	-	-	5.71	-	-	-	-	
	P		0.12		0.17				0.02					

Supplementary Table S5

CYP2C19*2 and *17 allele distribution according to ethnicity

		Ethnicity				
**Genotype**		Unknown	White	Black	Asian	**Other**
**CYP2C19*2**						
Missing or unknown	N	0	47	0	0	**0**
	%	-	3.1	-	-	-
Wild type	N	227	1031	7	151	**1**
	%	74	68	64	55	-
One allele	N	77	394	4	92	**0**
	%	25	26	36	33	-
Two alleles	N	4	34	0	33	**0**
	%	1.3	2.3	-	14	-
**CYP2C19*17**						
Missing or unknown	N	306	267	1	274	**1**
	%	99	18	9	99	-
Wild type	N	2	720	6	1	**0**
	%	<1	48	55	<1	-
One allele	N	0	440	4	1	**0**
	%	-	29	36	<1	-
Two alleles	N	0	79	0	0	**0**
	%	-	5.2	-	-	-
